# Exendin-4 enhances the differentiation of Wharton’s jelly mesenchymal stem cells into insulin-producing cells through activation of various β-cell markers

**DOI:** 10.1186/s13287-016-0374-4

**Published:** 2016-08-11

**Authors:** Dina H. Kassem, Mohamed M. Kamal, Abd El-Latif G. El-Kholy, Hala O. El-Mesallamy

**Affiliations:** 1 Biochemistry Department, Faculty of Pharmacy, Ain Shams University, Cairo, Egypt; 2 Gynecology and Obstetrics Department, Faculty of Medicine, Ain Shams University, Cairo, Egypt

**Keywords:** Wharton’s jelly, Mesenchymal stem cells, Exendin-4, Insulin-producing cells, Diabetes mellitus

## Abstract

**Background:**

Diabetes mellitus is a devastating metabolic disease. Generation of insulin-producing cells (IPCs) from stem cells, especially from Wharton’s jelly mesenchymal stem cells (WJ-MSCs), has sparked much interest recently. Exendin-4 has several beneficial effects on MSCs and β cells. However, its effects on generation of IPCs from WJ-MSCs specifically have not been studied adequately. The purpose of this study was therefore to investigate how exendin-4 could affect the differentiation outcome of WJ-MSCs into IPCs, and to investigate the role played by exendin-4 in this differentiation process.

**Methods:**

WJ-MSCs were isolated, characterized and then induced to differentiate into IPCs using two differentiation protocols: protocol A, without exendin-4; and protocol B, with exendin-4. Differentiated IPCs were assessed by the expression of various β-cell-related markers using quantitative RT-PCR, and functionally by measuring glucose-stimulated insulin secretion.

**Results:**

The differentiation protocol B incorporating exendin-4 significantly boosted the expression levels of β-cell-related genes *Pdx-1*, *Nkx2.2*, *Isl-1* and *MafA*. Moreover, IPCs generated by protocol B showed much better response to variable glucose concentrations as compared with those derived from protocol A, which totally lacked such response. Furthermore, exendin-4 alone induced early differentiation markers such as *Pdx-1* and *Nkx2.2* but not *Isl-1*, besides inducing late markers such as *MafA*. In addition, exendin-4 showed a synergistic effect with nicotinamide and β-mercaptoethanol in the induction of these markers.

**Conclusions:**

Exendin-4 profoundly improves the differentiation outcome of WJ-MSCs into IPCs, possibly through the ability to induce the expression of β-cell markers.

**Electronic supplementary material:**

The online version of this article (doi:10.1186/s13287-016-0374-4) contains supplementary material, which is available to authorized users.

## Background

Diabetes mellitus (DM) is a devastating metabolic disease associated with increased morbidity and mortality [1]. It is growing at an alarming rate, affecting more than 415 million people worldwide [2]. In DM, insulin-secreting β cells are damaged to different extents, leading to either absolute or relative insulin deficiency in type 1 and type 2 DM, respectively [1].

The transplantation of pancreatic islets has been demonstrated as a very effective treatment for DM, which could allow temporal insulin independence [3]. However, the availability of the donor islets could never meet the demand of the DM burden, which firmly establishes the clinical value of generating surrogate β cells from alternative renewable sources such as differentiation from stem cells [4].

Actually, within the last few years, various types of stem cells have been shown to be able to give rise to insulin-producing cells (IPCs), including embryonic stem cells (ESCs) [5, 6] and mesenchymal stem cells (MSCs) [7–9]. In fact, MSCs could indeed represent the stem cells of choice for regenerative medicine [10]. Among the various sources of MSCs, the umbilical cord (UC) together with other extra-embryonic tissues, which are routinely discarded at parturition, provide an untapped source of MSCs [11]. These sources do not impose any ethical concerns such as those which exist regarding ESCs, thus implying great potential for biomedical applications and cell-based therapeutic approaches [12].

The UC has been proved to be a good source of MSCs either from cord blood or cord tissue, also known as Wharton’s jelly (WJ) MSCs. The main fascination of WJ-MSCs lies in their possible banking, ease of isolation and large ex-vivo expansion capacity, as well as their demonstrated multipotency and immunomodulatory activities, which nominate them to become the new gold standard for MSC-based therapies [10]. Actually, in the context of diabetes research, WJ-MSCs sparked great interest [13] that is further encouraged by the recent interesting findings of multiple beneficial effects upon their injection into either diabetic patients or animals. Interestingly, Liu et al. applied WJ-MSC transplantation in type 2 DM patients, and demonstrated that treatment with WJ-MSCs can indeed improve metabolic control and β-cell function in patients with type 2 DM. They also suggested that their mechanism of action may have involved improvements in systemic inflammation [14]. Furthermore, another report highlighted the effect of intravenous infusion of human WJ-MSCs as a therapy by administering these cells in a type 2 DM rat model. The rats treated with WJ-MSCs exhibited increased numbers of β cells, suggesting the therapeutic potential of WJ-MSCs in β-cell regeneration [15]. Interestingly, our laboratory reported previously that WJ-MSCs exhibited better differentiation potential and control of hyperglycemia in streptozotocin-induced diabetic rats as compared with cord blood MSCs [16].

It is noteworthy here that there are several protocols used for generation of IPCs from MSCs in the literature. These protocols employ a huge variety of extrinsic factors [17, 18] and induction periods, which could vary from several days [9] to several months [17, 19]. Nevertheless, despite all of the vigorous efforts to generate mature functioning β cells from MSCs or even other types of stem cells, the majority of the obtained IPCs mostly fail to properly secrete insulin in response to glucose, and the reason for such anomalies together with the mechanisms through which differentiation occurs are far from complete elucidation [20].

Interestingly, the glucagon-like peptide-1 (GLP-1) receptor agonist exendin-4 [21] has been incorporated among the various inducing extrinsic factors for the differentiation of stem cells from various sources to IPCs [22–24]. Exendin-4 is a 39-amino-acid peptide which was initially isolated from the venom of lizard *Heloderma suspectum* [25]. Exendin-4 has been found to act as a long-acting GLP-1 receptor agonist which, like GLP-1, has been reported to stimulate both β-cell replication and neogenesis, resulting in increased β-cell mass and improved glucose tolerance [26].

However, the effects of exendin-4 on the differentiation of WJ-MSCs specifically have not been studied adequately. Given the unique transcriptomic profile of WJ-MSCs [27] and their increasingly important potential for regenerative medicine applications [28], optimizing efficient differentiation protocols for these cells is strongly warranted.

The purpose of this study was therefore to investigate the role of exendin-4 in the generation of IPCs from WJ-MSCs. In addition, we examined the effect of exendin-4 alone and in combination with other extrinsic factors on the expression of β-cell markers to gain more insight into the role played by exendin-4 in this differentiation process.

## Methods

### Isolation and culture of WJ-MSCs

All of the experiments were carried out in accordance with the approved guidelines and all of the procedures were approved by the ethical committees of both the Faculty of Pharmacy and the Faculty of Medicine, Ain Shams University, Cairo, Egypt. The UCs were obtained from the Gynecology and Obstetrics Department, Ain Shams University Hospitals, from both cesarean section and normal labor after obtaining signed informed consent from the parents. Fresh human UCs were collected in sterile phosphate-buffered saline (PBS), maintained in ice and processed within 1–4 hours post delivery. To avoid any chance for contamination, the collected UC was swabbed with 70 % alcohol for just a few seconds and then washed twice with sterile PBS. Afterwards, it was cut into smaller pieces (each 2–5 cm long). All isolation procedures were carried out under aseptic conditions. The cord blood vessels were removed and the UC WJ was processed until obtaining single cells by the explant method as described previously with few modifications [11, 29]. The WJ was cut into small pieces (5–10 mm) which were placed in six-well plates with complete low-glucose Dulbecco’s modified Eagle’s medium (LG-DMEM) supplied with 10 % FBS, 2 mM l-glutamine, 100 U/ml penicillin and 100 μg/ml streptomycin, and subsequently incubated in 37 °C, 5 % CO_2_ humidified atmosphere. Adherent fibroblast-like cells appeared after 10–14 days. These cells were subcultured using 0.05 % trypsin–EDTA, and medium was changed every other day.

### Immunophenotyping of WJ-MSCs

WJ-MSCs at the third passage were trypsinized and washed twice with PBS, and then 100,000 cells were incubated at 4 °C in the dark for 20 minutes with human monoclonal antibodies labeled with either fluroisothiocyanate (FITC) or phycoerythrin (PE) as follows: CD34 PE, CD14 PE (BD, Pharmingen), CD73 FITC, CD90 FITC, CD105 PE (Beckman Coulter, Marseille, France). Mouse isotype IgG_1_ FITC and PE antibodies were employed as controls. The cells were then washed and suspended in 500 μl of FACS buffer and analyzed by a CYTOMICS FC 500 Flow Cytometer (Beckman Coulter, FL, USA) using CXP Software version 2.2.

### Differentiation of WJ isolated cells into adipogenic, osteogenic and chondrogenic lineages

We performed adipogenic, osteogenic and chondrogenic differentiation using the Human Mesenchymal Stem Cell Functional Identification Kit (R&D Systems Inc., MN, USA). The induction processes for the three lineages were performed according to the manufacturer’s instructions. Noninduced control WJ-MSCs were fed with complete growth medium (10 % FBS LG-DMEM) on the same schedule of each investigated lineage. Regarding adipogenic differentiation, after about 7 days lipid vacuoles started to appear in the induced cells. The detection of the resultant differentiated cells was carried out using Oil Red staining (Sigma-Aldrich, USA). For the osteogenic lineage, cells changed from spindle shaped to cuboidal shaped during differentiation, and differentiation was confirmed by Alizarin Red-S staining (Sigma-Aldrich, USA) for the calcium-rich extracellular matrix. Finally, regarding chondrogenic induction, cells changed from spindle shaped to cuboidal shaped during differentiation, and differentiation was confirmed by Alcian 8GX blue staining (Sigma-Aldrich, USA) for sulfated proteoglycan.

### Pancreatic endocrine differentiation

After two to four passages, 1 × 10^6^ WJ-MSCs were induced to differentiate into IPCs using two protocols. The first protocol (A) was carried out as described previously with slight modifications [9]; cells were preinduced for 48 hours with 10 mmol/L nicotinamide (Sigma-Aldrich, USA) and 1 mmol/L β-mercaptoethanol (Sigma-Aldrich, USA) in 10 % FBS LG-DMEM, and then reinduced for another 24 hours with 10 mmol/L nicotinamide and 1 mmol/L β-mercaptoethanol in serum-free high glucose (HG)-DMEM.

The second protocol (B) started exactly as protocol A; the cells were preinduced for 48 hours with 10 mmol/L nicotinamide and 1 mmol/L β-mercaptoethanol in 10 % FBS LG-DMEM, and then reinduced for another 24 hours with 10 mmol/L nicotinamide and 1 mmol/L β-mercaptoethanol in serum-free HG-DMEM. However, this was followed by further induction for 7 days by 10 nmol/L exendin-4 (Sigma-Aldrich, USA) in serum-free HG-DMEM supplemented with 10 mmol/L nicotinamide and 1 mmol/L β-mercaptoethanol. Noninduced control WJ-MSCs were fed with complete growth medium (10 % FBS LG-DMEM) and kept for the same time as the differentiation protocol following the same culturing conditions as described earlier in this study.

### Effect of exendin-4 alone and in combination with other extrinsic factors on β-cell markers

Briefly, WJ-MSCs were cultured for 10 days in 5 % FBS HG-DMEM, supplemented with either 10 nmol/L exendin-4 alone, 10 nmol/L exendin-4 and 10 mmol/L nicotinamide, or 10 nmol/L exendin-4 together with 10 mmol/L nicotinamide and 1 mmol/L β-mercaptoethanol. Noninduced control WJ-MSCs were fed with complete growth medium (10 % FBS LG-DMEM) and kept for the same time as the induced cells following the same culturing conditions as described earlier in this study. RNA extraction and quantitative reverse transcriptase PCR (qRT-PCR) analyses were then performed on the resulting cells for various β-cell markers.

### RNA extraction and real-time RT-PCR analysis

Both control undifferentiated WJ-MSCs and differentiated IPCs (resulting from both protocols) were collected. RNA was isolated using TRIzol Reagent (Life Technologies, USA) according to the manufacturer’s instructions. Briefly, 3 × 10^6^ cells were treated by 1 ml TRIzol followed by extraction using chloroform and isopropanol. The cDNA was prepared by the Verso™ cDNA synthesis kit (Thermo Scientific, USA) using 0.5 μg RNA. Each qRT-PCR reaction was performed using 4 ng cDNA using the SYBR Green Master Mix (Applied Biosystems, USA). GAPDH was used as an internal control. ΔΔCt was used to calculate relative expression levels. RNA expression of various pancreatic development markers was measured by qRT-PCR. Forward and reverse primers for target genes are presented in Table 1. All qRT-PCR analyses were carried out using Step-One plus qRT-PCR (Applied Biosystems, USA).

**Table 1:** Forward and reverse primer sequences used for reverse transcriptase PCR

Gene	Forward primer	Reverse primer
*GAPDH*	GCCAAAAGGGTCATCATCTC	TGAGTCCTTCCACGATACCA
*Pdx-1*	GGAGCCGGAGGAGAACAAG	CTCGGTCAAGTTCAACATGACAG
*Nkx2.2*	TCTACGACAGCAGCGACAAC	TTGTCATTGTCCGGTGACTC
*Isl-1*	ATTTCCCTATGTGTTGGTTGCG	CGTTCTTGCTGAAGCCGATG
*MafA*	TTCAGCAAGGAGGAGGTCAT	CGCCAGCTTCTCGTATTTCT

### Functional assessment of differentiated cells by glucose challenge test for insulin release (GSIS assay)

The maturity of differentiated IPCs was assessed by its ability to secrete insulin in response to high glucose challenge. Briefly, the differentiated cells were washed twice with PBS and Krebs Ringer bicarbonate (KRB) buffer, and then incubated for 1 hour in KRB buffer supplemented with 5.5 mM glucose in 37 °C, 5 % CO_2_ humidified atmosphere. Afterwards, cells were incubated with 5.5 mM glucose for 1 hour and then with 16.7 mM glucose in KRB buffer in the same conditions for 1 hour, and the supernatant was collected at the end of each incubation and frozen at –80 °C until the time of the assay. Insulin release was detected by the Accubind® insulin enzyme-linked immunosorbent assay (Monobind Inc., CA, USA) according to the manufacturer’s instructions.

### Dithizone staining

About 1 × 10^5^ cells/well were seeded in six-well culture plates and exposed to various differentiation protocols. At the end of each differentiation protocol, Dithizone (DTZ) staining was performed to confirm IPC differentiation as described previously [30]. A stock solution of DTZ was prepared as follows: 50 mg of DTZ (Sigma Aldrich, USA) was completely dissolved in 5 ml of dimethyl sulfoxide (DMSO) and stored at –20 °C in the dark. For staining, a working solution was prepared by diluting the stock solution 1:100 in culture medium and then filtered through a 0.2-μm nylon filter. Then 3 ml of DTZ working solution was added to each well and incubated for at least 30 minutes at 37 °C. The cells were then carefully washed three times by PBS. Crimson red-stained IPCs were observed under an inverted phase-contrast microscope. Uninduced WJ-MSCs cultured in complete growth medium (LG-DMEM supplemented with 10 % FBS) were used as control.

### Statistical analyses

Data are presented as mean ± standard error of mean. Comparisons between the groups were conducted using a *t* test. For experiments with more than two groups comparison between means was conducted using one-way ANOVA, and the Bonferroni post-hoc test was applied to compare individual groups. All statistical analyses were carried out using the Windows-based SPSS statistical package (SPSS version 17.0; SPSS, Chicago, IL, USA). *p* < 0.05 was considered significant.

## Results

### WJ is a potential source of MSCs exhibiting all MSC characteristics

**Fig. 1:**
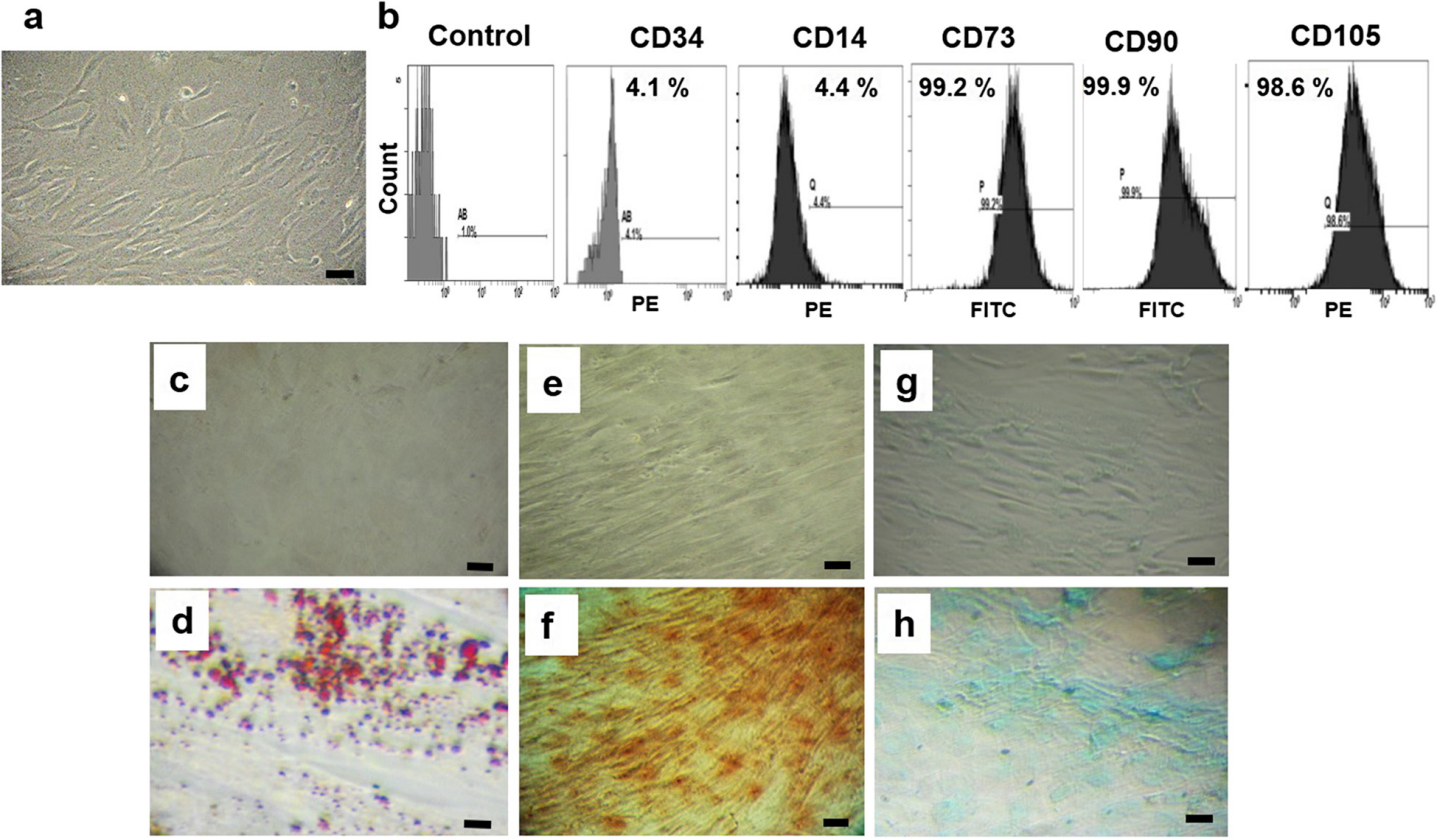
**a** Phase-contrast image of isolated WJ-MSCs (passage 3) showing homogeneous fibroblast-like cells. Magnification: 10×; *scale bar* = 100 μm. **b** Immunophenotyping for isolated WJ-MSCs; cells were labeled with FITC-conjugated or PE-conjugated antibodies and examined by flow cytometry. The immunophenotyping profile of WJ-MSCs showed negative for CD34 and CD14 but positive for CD73, CD90 and CD105. **c**–**h** Differentiation of isolated cells down several mesenchymal lineages: **c** uninduced WJ-MSCs as control for adipogenic differentiation; **d** induced WJ-MSCs showing red staining of oil droplets using Oil Red, characteristic for successful adipogenic differentiation; **e** uninduced control WJ-MSCs for osteogenic differentiation experiment; **f** induced WJ-MSCs showing positive Alizarin Red-S staining for calcium-rich extracellular matrix, indicating successful osteogenic differentiation characteristic for MSCs; **g** uninduced control WJ-MSCs for the chondrogenic differentiation experiment; **h** induced WJ-MSCs showing blue staining by Alcian 8GX blue for sulfated proteoglycan, indicating the successful chondrogenic differentiation of isolated WJ-MSCs. *Scale bar* = 100 μm

Adherent cells with fibroblast-like morphology could be observed as early as 10–14 days post plating of the explants of UC WJ. As shown in Fig. 1a, these cells were almost homogeneous, resembling MSC morphology. These cells showed excellent culture properties. Furthermore, these isolated fibroblast-like cells exhibit all of the MSC characteristics defined by the International Society for Cellular Therapy (ISCT) [31]. First, they were plastic adherent. Second, we performed flow cytometry for MSCs and hematopoietic specific cluster of differentiation (CD) markers. As shown in Fig. 1b, immunophenotyping revealed that isolated cells were negative for CD34 (hematopoietic stem cells) and CD14 (monocytes); the percentage of cells expressing these CDs did not exceed 5 %. On the contrary, they were positive for MSC markers CD73, CD90 and CD105, exhibiting expression intensities of 99.2 %, 99.9 % and 98.6 % respectively for these markers. These results indicate a relatively homogeneous mesenchymal phenotypic population of WJ-MSCs.

As a functional assay to further confirm the MSC identity of isolated cells, we examined the differentiation potential of these cells towards mesenchymal lineages. As shown in Fig. 1c–h, these WJ-MSCs exhibit adipogenic differentiation potential; detected by Oil Red staining of lipid droplets in comparison with control undifferentiated cells. Furthermore, these cells show an osteogenic differentiation potential; detected by Alizarin Red-S staining for calcium-rich extracellular matrix as compared with their control uninduced counterparts. Finally, the chondrogenic differentiation potential of the isolated WJ-MSCs was detected by Alcian 8GX blue staining for sulfated proteoglycan in comparison with control uninduced cells.

### Exendin-4 improves the differentiation outcome of WJ-MSCs to IPCs

**Fig. 2:**
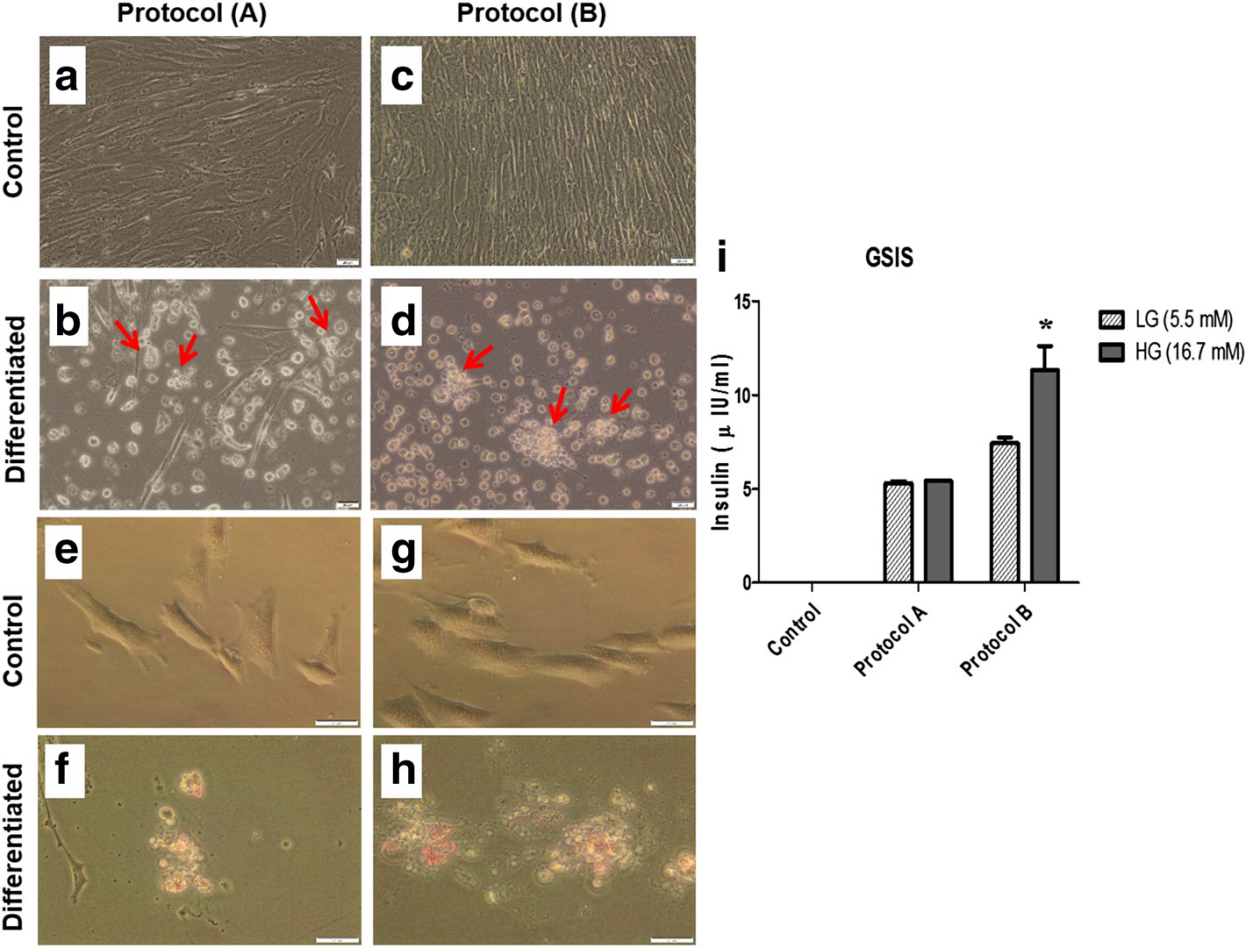
**a**–**d** Phase-contrast images of differentiated IPCs obtained from WJ-MSCs after induction by differentiation protocols: upon differentiation, cells lose their fibroblastic morphology and tend to aggregate forming clusters, which tend to detach and grow in suspension media, in contrast to their control WJ-MSCs which retain fibroblast-like morphology. Magnification, 20×; *scale bar* = 20 μm. **e**–**h** Phase-contrast images of generated IPCs stained by Dithizone (DTZ): **e** uninduced WJ-MSCs as a control showing negative DTZ staining; **f** IPCs generated by protocol A showing positive crimson red staining by DTZ; **g** uninduced WJ-MSCs as a control showing negative DTZ staining; **h** IPCs generated by protocol B showing positive crimson red staining by DTZ. Magnification 40×; *scale bar* = 20 μm. **i** In-vitro GSIS assay for differentiated IPCs derived from protocol A (without exendin-4) and protocol B (with exendin-4); insulin release in response to either 5.5 mM or 16.7 mM glucose concentrations was measured after 1 hour of incubation. It is noteworthy that control uninduced undifferentiated WJ-MSCs did not show any detectable levels of secreted insulin in response to either 5.5 mM or 16.7 mM glucose concentrations. Results presented as mean ± standard error of mean, obtained from three independent experiments. *Means are significantly different from secreted insulin levels in response to 5.5 mM glucose at *p <* 0.05. *GSIS* glucose-stimulated insulin secretion, *HG* high glucose, *LG* low glucose

For generation of IPCs from WJ-MSCs, we tried two protocols: protocol A without exendin-4; and protocol B with exendin-4. As shown in Fig. 2a–d, at the end of both protocols the cells tended to lose their fibroblast-like shape and formed aggregates. Furthermore, some cells started to detach and grew as a suspension in culture media. However, control undifferentiated cells for both protocols kept their fibroblast-like morphology throughout the whole protocol period. Interestingly, when staining the resulting putative IPCs with DTZ, the resulting differentiated cells from both protocols were positively stained by DTZ as shown in Fig. 2e–h.

To functionally assess the IPCs generated with these protocols, we performed glucose-stimulated insulin secretion (GSIS) for the derived IPCs. As shown in Fig. 2i, IPCs generated by protocol A did not show any response to variable glucose concentrations (LG 5.5 mM: 5.3 ± 0.12 μIU/ml secreted insulin, HG 16.7 mM: 5.43 ± 0.01 μIU/ml secreted insulin, *p* = 0.339). However, IPCs generated by protocol B showed higher secretion of insulin in response to HG as compared with LG concentrations (LG 5.5 mM: 7.45 ± 0.3 μIU/ml secreted insulin, HG 16.7 mM: 11.36 ± 1.23 μIU/ml secreted insulin, *p* = 0.037). So far, these results indicate better differentiation of IPCs generated by protocol B. Moreover, we examined the expression of *Pdx-1* in differentiated IPCs generated from WJ-MSCs using the two protocols by immunocytochemistry. As shown in Additional file 1: Figure S1 (uppermost panel), control undifferentiated cells obviously lacked *Pdx-1* expression. Upon exposure of these cells to protocol A (middle panel) they failed to express *Pdx-1*, indicating failure of this protocol to induce proper differentiation. On the contrary, using protocol B (lowest panel) the cells showed expression of *Pdx-1*, indicating differentiation of these cells into IPCs.

**Fig. 3:**
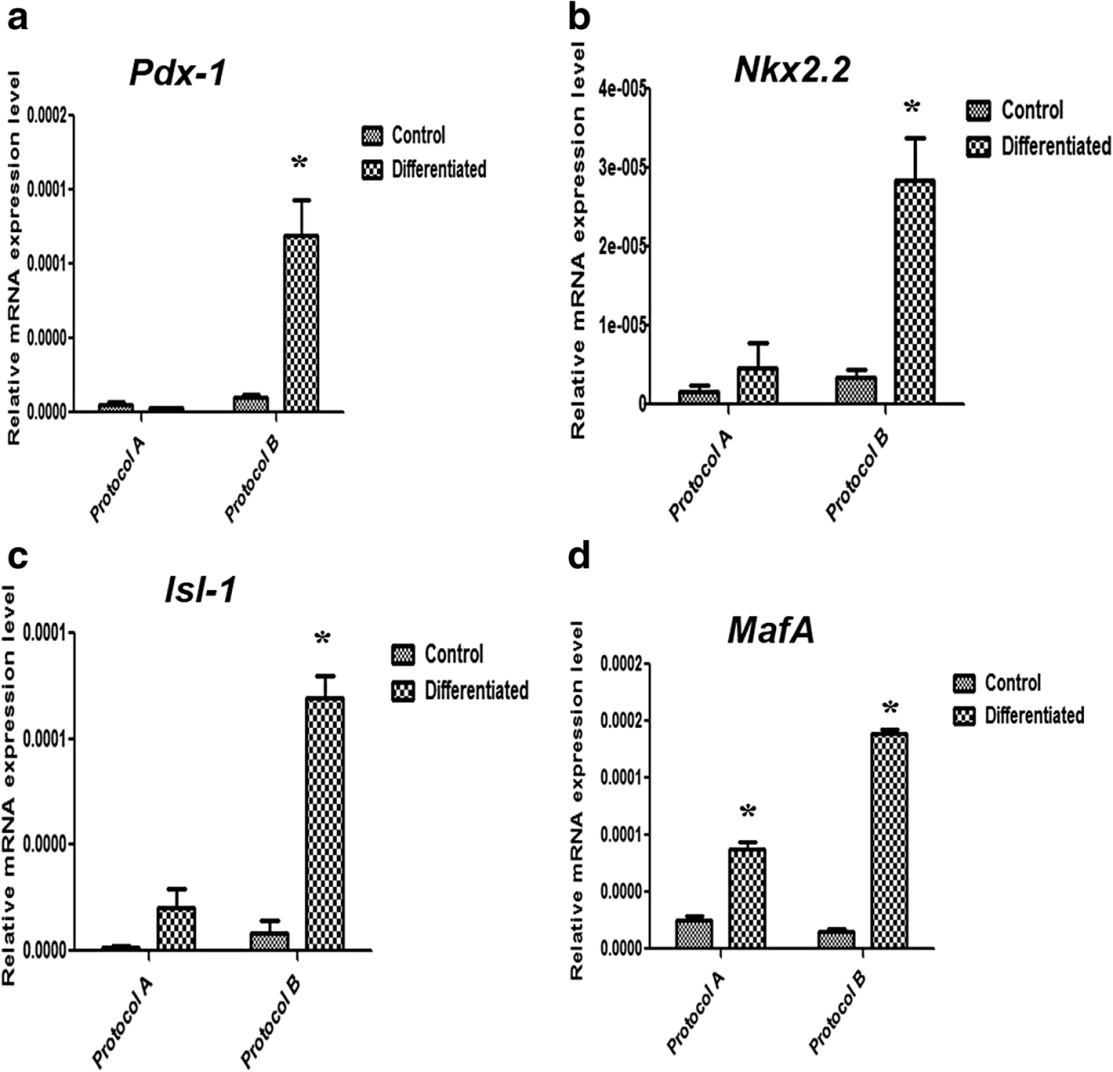
Gene expression analyses by qRT-PCR for several β-cell markers. **a** *Pdx-1*, **b** *Nkx2.2*, **c** *Isl-1* and **d** *MafA* in differentiated IPCs derived from differentiation protocols A and B as compared with their control uninduced WJ-MSCs. Results are presented as mean ± standard error of mean, obtained from three independent experiments. *Means are significantly different from control uninduced WJ-MSCs at *p* < 0.05

In spite of similar morphological changes, the mRNA expression levels of β-cell-related genes were quite different between these protocols. As shown in Fig. 3a–d, the induced cells derived from protocol A showed relatively no elevation at all for *Pdx-1* expression, a slight modest elevation of the expression levels of *Nkx2.2* and *Isl-1*, and elevation of *MafA* transcript levels. However, for protocol B the induced cells showed a profound significant elevation for the expression levels of *Pdx-1*, *Nkx2.2*, *Isl-1* and *MafA*, as compared with their control uninduced counterparts.

### Exendin-4 alone can induce *Pdx-1*, *Nkx2.2* and *MafA* but not *Isl-1*, while exhibiting a synergistic effect with nicotinamide and β-mercaptoethanol

**Fig. 4:**
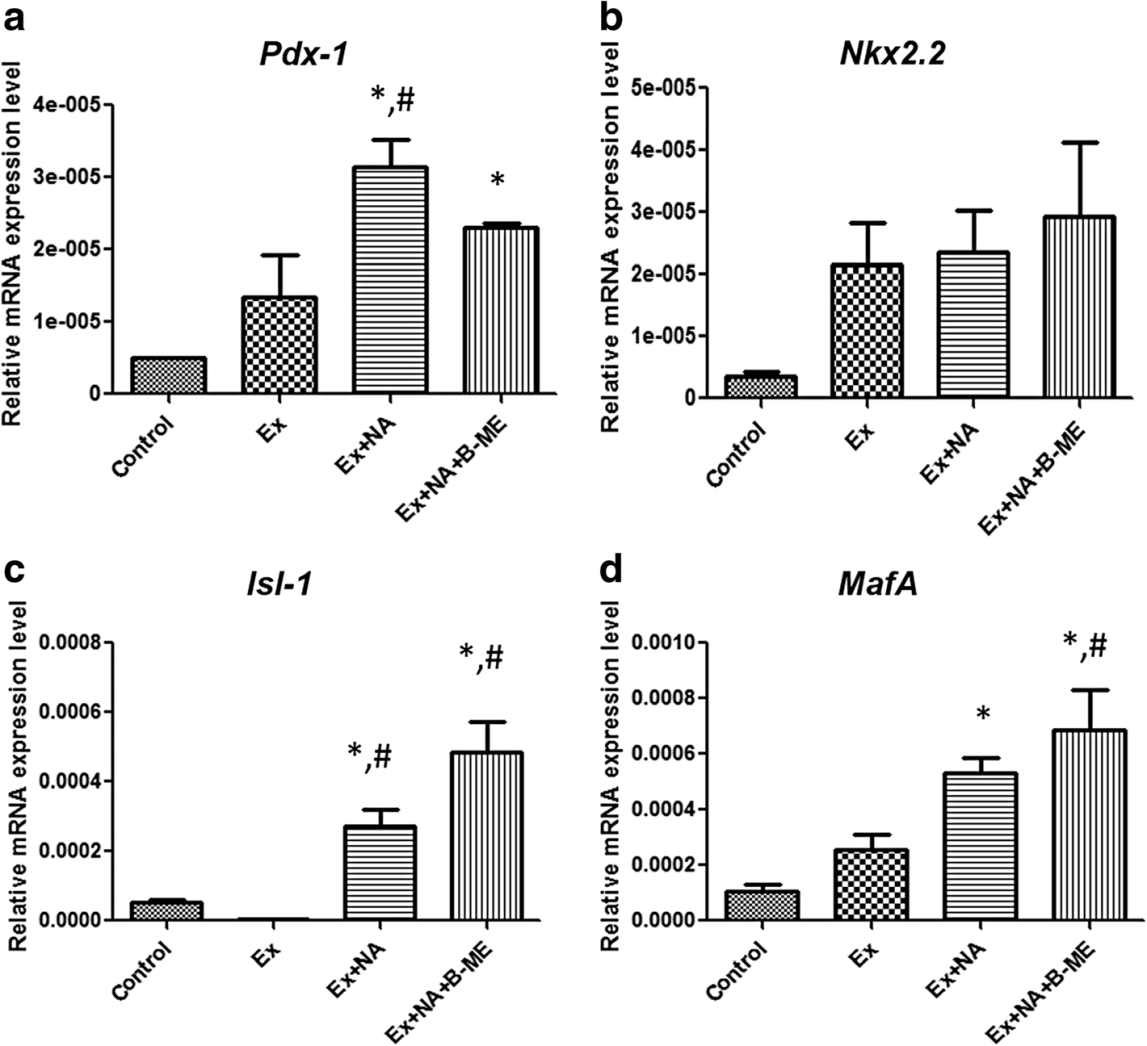
Gene expression analyses by qRT-PCR for cells induced by individual extrinsic factors, namely exendin-4 alone (*Ex*), exendin-4 plus nicotinamide (*Ex + NA*), and exendin-4 plus nicotinamide and β-mercaptoethanol (*Ex + NA + BME*), in 5 % FBS HG-DMEM. Gene expression analyses were done for several β-cell-related genes, namely **a** *Pdx-1*, **b** *Nkx2.2*, **c** *Isl-1* and **d** *MafA* presented as relative mRNA expression levels in induced cells relative to their uninduced control counterparts. Results presented as mean ± standard error of mean for three independent experiments. *Means are significantly different from control at *p* < 0.05. ^#^Means are significantly different from exendin-4 alone (Ex) at *p* < 0.05

To gain more insight into the role played by exendin-4 alone and in combination with other extrinsic factors on the differentiation markers of WJ-MSCs into IPCs, we incubated the cells with exendin-4 alone and in combination with nicotinamide alone or nicotinamide plus β-mercaptoethanol. As shown in Fig. 4a, exendin-4 alone induced the *Pdx-1* expression level. This elevation was further induced by adding nicotinamide and β-mercaptoethanol to significant levels as compared with the control group. Also, as shown in Fig. 4b, exendin-4 showed an induction of *Nkx2.2* transcript level as compared with the control group. However, in contrast to *Pdx-1*, addition of nicotinamide and β-mercaptoethanol did not show any further elevation in the expression levels of this transcription factor. Interestingly, Fig. 4c clearly illustrated that exendin-4 alone failed to induce the expression of *Isl-1*, while adding nicotinamide and β-mercaptoethanol with exendin-4 induced increased expression levels of *Isl-1*. Again, as shown in Fig. 4d, the *MafA* expression level showed a slight elevation with exendin-4 alone. However, addition of nicotinamide and β-mercaptoethanol with exendin-4 significantly induced further elevation of *MafA* levels as compared with the control group.

## Discussion

In the current study, we isolated WJ-MSCs and investigated their differentiation into IPCs using two differentiation protocols; with or without exendin-4. Our results showed that WJ-MSCs indeed fulfilled all MSC characteristics, and showed good potential to differentiate into IPCs. Additionally, exendin-4 was found to have profound beneficial effects on the differentiation outcome of those WJ-MSCs into IPCs. Besides, although our results showed that exendin-4 alone can induce the expression of some β-cell markers, namely *Pdx-1*, *Nkx2.2* and *MafA* but not *Isl-1*, there still exists a synergistic effect on the induction of such markers upon addition of nicotinamide and β-mercaptoethanol with overall improvement of differentiation outcome.

Strategies to create surrogate β cells for therapeutic replacement have ignited significant excitement over the last decade, which has fueled profound interest in deriving functioning IPCs from stem cells [32]. Our results regarding the observed ease of isolation and relatively homogeneous population of isolated WJ-MSCs came in accordance with all of the reports highlighting the important potential of WJ-MSCs for various regenerative medicine applications [28].

Despite all efforts so far regarding the differentiation of stem cells to IPCs, current protocols are not optimized for different reasons, including the pleotropic effects induced by individual morphogens, together with the complexity of the signaling pathways involved and differences between the various types of used stem cells [18]. Further exploration into the differentiation of several stem cells to IPCs is thus indeed warranted together with investigating the various effects of incorporated inducing factors in the differentiation process [17, 33].

In the current study, we investigated two differentiation protocols, namely protocol A (without exendin-4) and protocol B (with exendin-4). Actually, both protocols induced the cells to detach and form clusters, and both of them showed positive DIZ staining; no apparent morphological differences were thus observed in IPCs generated from both protocols. Interestingly, the IPCs derived from protocol B, with exendin-4, showed a profound induction for the expression levels of all investigated β-cell-related genes in the current study, *Pdx-1*, *Nkx2.2*, *Isl-1* and *MafA*, as compared with their control uninduced WJ-MSCs. These beneficial effects were further extended to our observations in the GSIS assay in which IPCs generated by protocol B demonstrated a reasonable response to HG as compared with LG concentrations, while those generated from protocol A totally lacked such response. Moreover, when carrying out immunocytochemistry to detect *Pdx-1* in IPCs generated by both protocols, those generated by protocol B clearly expressed *Pdx-1* but those derived from protocol A did not show such clear expression. Those findings also imply failure of protocol A lacking exendin-4 to induce proper differentiation, and further highlight the beneficial effects of exendin-4 on the differentiation outcome. Moreover, exendin-4 could enhance the viability of the generated IPCs as shown in Additional file 1: Figure S2. Actually, this might be attributed to the beneficial effects which have been reported recently for exendin-4 regarding improving MSC proliferation and cell survival [34].

Our findings regarding the enhancement of differentiation of WJ-MSCs into IPCs under the effect of exendin-4 are in agreement with previous studies which reported beneficial effects of exendin-4 for the differentiation of ESCs and increasing the insulin release by IPCs [22], MSCs from adipose tissue [35] or BM-MSCs [36].

Interestingly, apart from its beneficial effects on IPC differentiation, exendin-4 was previously reported to increase β-cell mass by stimulating β-cell proliferation and neogenesis in diabetic rats [26]. However, the exact mechanism of action of exendin-4 during the differentiation process and even its anti-diabetic actions or effects on β cells in clinical settings (exenatide) are not understood completely [37]. Among these suggested mechanisms are the induction of *Pdx*-1, and acting through several intracellular pathways such as the phosphatidylinositol-3-kinase (PI3K), the hedgehog or the MAPK/ERK pathway [18]. The suggested mechanism through inducing *Pdx*-1 was exhibited in our study by the significantly elevated expression of *Pdx*-1 transcript in IPCs generated from protocol B as compared with its very low expression levels in those obtained from protocol A.

Although exendin-4 has been implicated extensively in generation of IPCs from various forms of MSCs including BM-MSCs [36], adipose MSCs [38] and even WJ-MSCs [13], the exact mechanism of exendin-4 on differentiation of MSCs into IPCs is far from complete elucidation and studies involved in understanding this mechanism are scarce. Accordingly, we decided to study the effect of exendin-4 alone and in combination with nicotinamide and β-mercaptoethanol in this differentiation process.

Interestingly, our results clearly demonstrated different effects of exendin-4 on various β-cell differentiation markers. First, we found that exendin-4 can alone induce *Pdx-1*. Moreover, adding nicotinamide and β-mercaptoethanol caused further induction of this transcription factor. Several reports have shown that exendin-4 upregulated *Pdx-1* during β-cell regeneration and that the effect of exendin-4 on β cells is mediated by *Pdx-1* [39]. Actually, *Pdx-1* is important for early pancreatic development and formation of β cells, and also acts as an insulin secretion activator [40]. Moreover, overexpression of *Pdx-1* has been implicated in the differentiation of ESCs into IPCs [41]. Our results therefore imply that the improvement of differentiation outcome induced by exendin-4 might be mediated by *Pdx-1*.

In addition to *Pdx-1* as an early pancreatic development factor, exendin-4 also induced the expression of *Nkx2.2* transcription factor in WJ-MSCs. *Nkx2.2* is implicated in early pancreatic development and, again, its overexpression induced β-cell differentiation from ESCs [42, 43]. In contrast to *Pdx-1* and *Nkx2.2*, exendin-4 failed alone to induce *Isl-1*, another transcription factor implicated in later endocrine differentiation during pancreatic development [44]. However, when exendin-4 was added with nicotinamide and/or β-mercaptoethanol, all of them showed some sort of synergism and induced the expression of *Isl-1* significantly as compared with control noninduced cells and as compared with induction using exendin-4 alone. Collectively, these results indicate that exendin-4 can be playing a role in the early development of IPCs from MSCs through its effect on *Pdx-1* and *Nkx2.2*, and possibly through *Isl-1* in the presence of nicotinamide and β-mercaptoethanol, but not alone. We assume this is considered an interesting finding that confirms not only the need for several factors to achieve complete differentiation to IPCs, but also reflects the complicated steps of such a process.

Interestingly, our results demonstrated that *MafA* transcript levels could be induced by exendin-4. *MafA* controls and activates the expression and secretion of insulin [45]. It is noteworthy here that exendin-4 at the dose used in our protocols was reported previously to increase the insulin secretion by β cells differentiated from ESCs [22]. Moreover, a recent study showed that proper activation of *MafA* at a specific stage of β-cell differentiation is critical for achieving optimum maturation of β cells [46]. Actually, the induced expression of *MafA* in the current study was enhanced with the addition of nicotinamide and β-mercaptoethanol, indicating a synergistic effect of these three factors for better differentiation of WJ-MSCs into IPCs, with the ultimate goal of attaining fully differentiated IPCs. This was clearly reflected in the far better GSIS of the IPCs generated from protocol B, with exendin-4, than that of protocol A, as well as the better expression of Pdx-1 protein in IPCs generated from protocol B as compared with those derived from protocol A. Collectively, these results clearly demonstrate the importance of exendin-4 in attaining better mature IPCs from WJ-MSCs and, therefore, improving the differentiation outcome of these cells.

It is noteworthy here that nicotinamide and β-mercaptoethanol used together in protocol A could induce some transcription factors, namely *Nkx2.2*, *Isl-1* and *MafA*. On the contrary, nicotinamide and β-mercaptoethanol showed a synergistic effect with exendin-4 in induction of *Pdx-1*, *Isl-1* and *MafA*. From these results one can assume that these extrinsic factors are acting in a complementary way in the induction of the complex process of differentiation. Accordingly, one can recommend, with confidence, using these extrinsic factors together in order to achieve better functioning IPCs. Also, we can portray exendin-4 as an indispensable factor in the differentiation protocols for WJ-MSCs into IPCs based on the levels of induction of β-cell markers in protocol B and better functionality reflected in better GSIS of this protocol.

Collectively, it seems that exendin-4 can induce several stages in the complex differentiation process, including the early stages exemplified by *Pdx-1* and *Nkx2.2*, in addition to late stages of insulin secretion exemplified by *MafA*.

This study is one of the few that dissect the role of exendin-4 on various β-cell markers upon differentiation of WJ-MSCs. This will be of great help in understanding the mechanism behind the effect of exendin-4 on the generation of IPCs from MSCs of different sources. Interestingly, a recent study by Xin et al. [47] showed that IPCs differentiated from human BM-MSCs could ameliorate diabetes in streptozotocin-induced diabetic nude mice. This implicates that differentiated cells can find their ways into diabetes regenerative medicine. However, the existing induction strategies of IPCs from MSCs need to be modified and improved. Our study is a step toward better understanding and developing a competent protocol of differentiation that ensures better functioning IPCs through the utilization of exendin-4 side by side with nicotinamide and β-mercaptoethanol.

In fact, the synergistic effect between exendin-4 with nicotinamide and β-mercaptoethanol observed in the induction of expression of several β-cell markers like *Pdx-1*, *Isl-1* as well as *MafA* sheds lights on the fact that the effects of exendin-4 could indeed be affected greatly by other extrinsic factors. This opens the door for a wide array of further investigations to study possible interactions between exendin-4 and other extrinsic factors incorporated during the course of various differentiation protocols in the literature, with the ultimate goal of achieving fully mature functional β cells for cell therapy of diabetes.

Our results demonstrate that exendin-4 greatly enhanced the differentiation of WJ-MSCs into IPCs. In doing so, exendin-4 induced several β-cell markers with some synergy with nicotinamide and β-mercaptoethanol. This is considered a step forward toward better understanding the differentiation process and achieving better functioning IPCs from MSCs that can be of value in diabetes regenerative medicine.

## Conclusions

Our results show that WJ-MSCs represent a readily available, noninvasive, highly promising source of stem cells for β-cell replacement therapies. Although the abundance of literature suggests that generation of IPCs from stem cells is feasible, many considerations such as the cell source, induction protocols and mechanisms of differentiation should be further explored before the application of these cells to clinical settings for treatment of DM. Most importantly, the principal novel finding of the current study is the beneficial enhancing effects of exendin-4 for the differentiation of WJ-MSCs into IPCs. In addition, this study shows that exendin-4 could induce early and late markers along the differentiation process. Finally, these findings open the door for further warranted investigations to gain more in-depth understanding of some hidden parts of the story of differentiation of stem cells into IPCs, and the mechanisms of action of various extrinsic factors. These hidden parts, if unraveled, will certainly help to resolve the difficulties of obtaining mature functioning IPCs.

## Abbreviations

CD, cluster of differentiation; DIZ, Dithizone; DM, diabetes mellitus; DMEM, Dulbecco’s modified Eagle’s medium; ESC, embryonic stem cell; FITC, fluroisothiocyanate; GSIS, glucose-stimulated insulin secretion; HG, high glucose; IPC, insulin-producing cell; ISCT, International Society for Cellular Therapy; Isl-1, insulin gene enhancer protein (ISLET-1); KRB, Krebs Ringer bicarbonate; LG, low glucose; MafA, v-maf avian musculoaponeurotic fibrosarcoma oncogene homolog A; MSC, mesenchymal stem cell; NKx2.2, homeobox protein Nkx2.2; Pdx-1, pancreatic and duodenal homeobox 1; PE, phycoerythrin; qRT-PCR, quantitative reverse transcriptase PCR; UC, umbilical cord; WJ, Wharton’s jelly

## Additional file

Additional file 1: Figure S1. Immunocytochemistry of control undifferentiated cells (upper most panel) and differentiated IPCs generated by both protocols; protocol A (middle panel) and protocol B (lowest panel) for Pdx-1 (red). The nuclei were stained with DAPI (blue), scale bar = 100 μm. **Figure S2.** Viability assay for control WJ-MSCs, differentiated IPCs in presence of exendin-4 and differentiated IPCs in absence of exendin-4. *significantly different from control at *p* < 0.05, ^#^significantly different from exendin-4 group at *p* < 0.05. (DOCX 186 kb)
