# Supplementary material for: Exendin-4 enhances the differentiation of Wharton’s jelly mesenchymal stem cells into insulin-producing cells through activation of various β-cell markers
Source: Stem Cell Res Ther. 2016 Aug 11;7:108. doi: 10.1186/s13287-016-0374-4 (PMC4981957; doi:10.1186/s13287-016-0374-4)
Supplement: Additional file 1: Figure S1. — Immunocytochemistry of control undifferentiated cells (upper most panel) and differentiated IPCs generated by both protocols; protocol A (middle panel) and protocol B (lowest panel) for Pdx-1 (red). The nuclei were stained with DAPI (blue), scale bar = 100 μm. Figure S2. Viability assay for control WJ-MSCs, differentiated IPCs in presence of exendin-4 and differentiated IPCs in absence of exendin-4. *significantly different from control at p < 0.05, #significantly different from exendin-4 group at p < 0.05. (DOCX 186 kb) [file 13287_2016_374_MOESM1_ESM.docx]

**Supplementary methods and figures**

**Immunocytochemistry:**

WJ-MSCs cells at passage 3 were collected, counted and 50,000 cells were seeded on glass coverslips. Cells were differentiated using induction protocols A and B as described earlier together with control cells cultured in complete growth medium without induction of differentiation. At the end of the differentiation protocol, cells were fixed by 4% paraformaldehyde (Sigma, USA) in PBS for 15 minutes followed by 3 washes with PBS, 5 minutes each. Then cells were blocked and permeabilized using 0.1% Triton-X in 4% serum. Afterwards, cells were incubated with primary antibody goat anti-human Pdx-1 (R&D, USA); dilution 1:300 at 4^o^C overnight. Afterwards, cells were washed and incubated with Alexafluor® 555 (Red fluorescence) secondary antibody (Life Technologies, USA). After washing, coverslips were mounted on a glass slide with a drop of the mounting medium SlowFade® gold antifade mountant with DAPI (Life technologies, USA) to counter stain the nuclei. Slides were photographed using a Nikon Fluorescence microscope controlled with NS Elements software, followed by deconvolution of the obtained images using AutoQuant software.

In order to examine the expression of Pdx-1 in differentiated IPCs generated from WJ-MSCs at protein level, we performed immunocytochemistry for Pdx-1 in control undifferentiated and differentiated cells generated from both protocols used in this study. As shown in **Figure S1** (the uppermost panel), control undifferentiated cells obviously lacked Pdx-1 expression. Upon exposure of these cells to protocol A (the middle panel), they failed to express Pdx-1 indicating failure of this protocol to induce proper differentiation. On the other hand, protocol B (lowest panel), the cells showed expression of Pdx-1 indicating differentiation of these cells into IPCs.

**
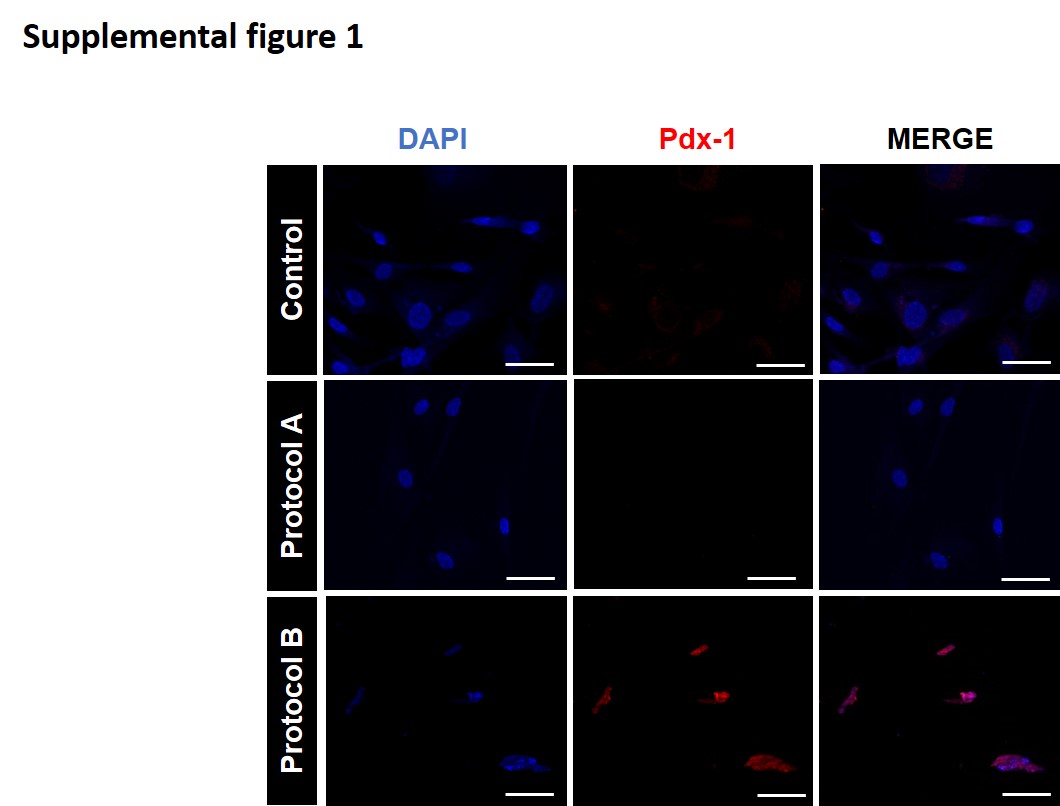
**

**Figure S1:** Immunocytochemistry of control undifferentiated cells (upper most panel) and differentiated IPCs generated by both protocols; protocol A (middle panel) and protocol B (lowest panel) for Pdx-1 (red). The nuclei were stained with DAPI (blue), scale bar = 100 µm.

**Viability assay using Trypan blue exclusion test:**

The viability assay was done using trypan blue exclusion test as described previously [[1](#_ENREF_1)]. Briefly, 1x10^5^cells/well were seeded in 6-well plate and treated as follows: control uninduced cells were kept in complete LG-DMEM/10% FBS for 10 days, the cells differentiated following protocol B using NA, β-ME and exendin-4 for 10 days (with exendin-4 in the graph) and the cells generated from protocol A but kept for extra 7 days in serum free HG-DMEM supplemented with NA and β-ME without exendin-4 (without exendin-4 in the graph). At the end of the experiments, cells were collected, stained with trypan blue and counted in 3 different fields.

As shown in **Figure S2**, the percentage viability of the IPCs generated from cells differentiated in presence of exendin-4 was slightly less than those of control group. However, viability was diminished in cells differentiated without exendin-4 for (Control: 86.25±0.072%, cells differentiated in presence of exendin-4: 76.6 ± 1.3 %, cells differentiated without exendin-4: 31.6 ± 3.4 %).


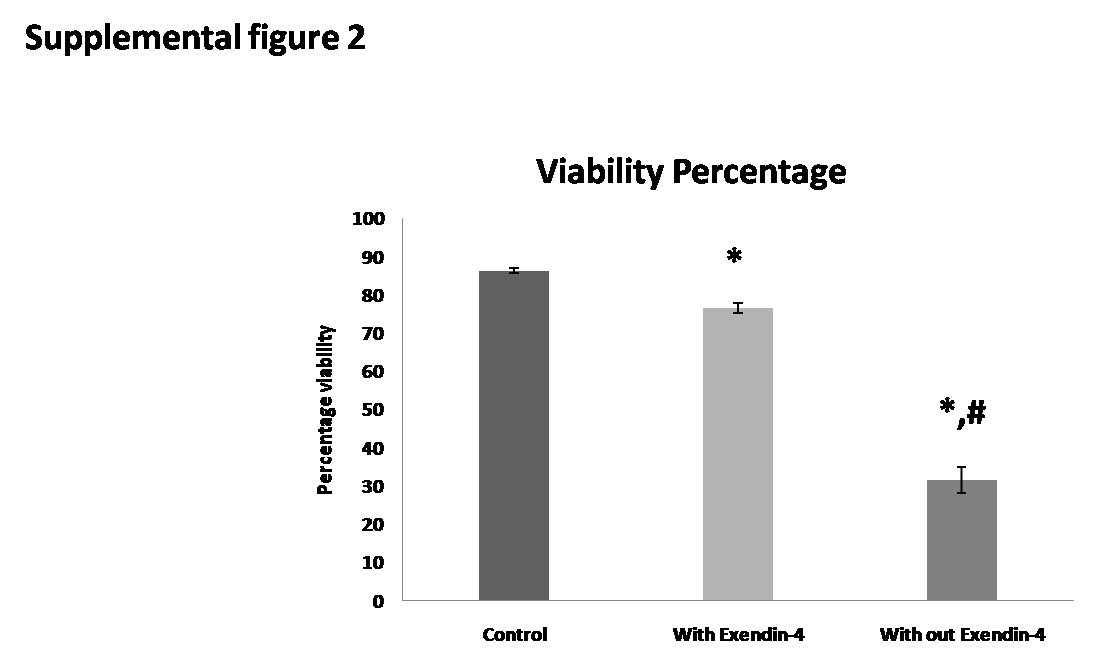


**Figure S2:** Viability assay for control WJ-MSCs, differentiated IPCs in presence of exendin-4 and differentiated IPCs in absence of exendin-4.

*: significantly different from control at p<0.05

#: significantly different from exendin-4 group at p<0.05

**References**

1. Strober W: Trypan blue exclusion test of cell viability. Curr Protoc Immunol*.* 2001;Appendix 3:Appendix 3B.
